# The “histological replacement growth pattern” represents aggressive invasive behavior in liver metastasis from pancreatic cancer

**DOI:** 10.1002/cam4.2954

**Published:** 2020-03-05

**Authors:** Kazuo Watanabe, Shuichi Mitsunaga, Motohiro Kojima, Hidetaka Suzuki, Ai Irisawa, Hideaki Takahashi, Mitsuhito Sasaki, Yusuke Hashimoto, Hiroshi Imaoka, Izumi Ohno, Masafumi Ikeda, Tetsuo Akimoto, Atsushi Ochiai

**Affiliations:** ^1^ Department of Hepatobiliary and Pancreatic Oncology National Cancer Center Hospital East Kashiwa Japan; ^2^ Course of Advanced Clinical Research of Cancer Juntendo University Graduate School of Medicine Tokyo Japan; ^3^ Division of Biomarker Discovery Exploratory Oncology Research and Clinical Trial Center National Cancer Center Hospital East Kashiwa Japan; ^4^ Division of Pathology Exploratory Oncology Research and Clinical Trial Center National Cancer Center Hospital East Kashiwa Japan; ^5^ Department of Pharmacy National Cancer Center Hospital East Kashiwa Japan; ^6^ Department of Analytical Biochemistry Meiji Pharmaceutical University Tokyo Japan; ^7^ Department of Radiation Oncology National Cancer Center Hospital East Kashiwa Japan

**Keywords:** liver metastasis, pancreatic cancer, prognostic factor, replacement growth pattern, tumor microenvironment

## Abstract

**Background:**

In the case of liver metastasis (LM), tumors showing the replacement growth pattern (RGP), in which metastatic cells infiltrate and replace hepatocytes with minimal desmoplastic reaction and inflammatory cell infiltration, associate with a poor prognosis. The heterogeneity, frequency, and prognostic value of the RGP in LM from pancreatic cancer (PCa) are not well known.

**Methods:**

In the circumference of treatment‐naïve resected LMs from patients with PCa, the heterogeneity of the GP was assessed. Next, the clinicopathological features of LMs showing the RGP in needle biopsy specimens were investigated in patients with treatment‐naïve advanced PCa.

**Results:**

Thirteen of the 14 (93%) in all resected LMs and 7 of the 9 (78%) in RGP component GP in resected LMs showed homogeneous GP. A RGP was found in 50% of the needle biopsy specimens of LMs obtained from 107 patients. The median overall survival times in the RGP group and non‐RGP group were 3.6 and 10.4 months. Multivariate analysis identified RGP as an independent poor prognostic factor. Median value of CD8 positive percentage in RGP was lower than that in non‐RGP (0.75 vs 1.46, *P* = .04). Median overall survival times in low CD8 groups tend to be shorter than those in high CD8 group (8.2 vs 4.2 months).

**Conclusion:**

Most LMs from PCa show a homogeneous GP. The RGP was observed in about a half of the LMs from PCa patients, and was identified as a poor prognostic factor.

## Graphical abstract

Morphological aspects of the tumor‐liver interface of liver metastasis, which was called growth pattern have been carefully studied in various cancers but not in pancreatic cancer. The RGP was observed in about a half of the LMs from PCa patients, and was identified as a poor prognostic factor.

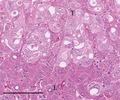

## 1 INTRODUCTION

The liver is a major site of metastasis from pancreatic cancer (PCa), and liver metastasis (LM) is associated with a poor prognosis.^1^, ^2^ Metastatic tumors, including LMs from PCa, are considered as desmoplastic tumors^3^ when the primary tumor has a highly fibrotic stroma. Recently, morphological aspects of the tumor‐liver interface of LMs have been carefully studied in various cancers^4^, ^5^ and the growth patterns (GPs) have been mainly classified into three types: the desmoplastic GP, the replacement GP, and the pushing type of GP.^6^ The desmoplastic GP is interpreted as an angiogenic GP and shows a better response to bevacizumab in cases with colorectal cancer.^7^ Antidesmoplastic therapy is a novel strategy for the treatment of PCa.^8^ Most nondesmoplastic tumors are classified as showing the replacement GP (RGP). RGP is defined as tumor cells that replace the nearby hepatocytes without destruction of the trabecular architecture of the liver, with minimal desmoplastic reaction and inflammatory cell infiltration.^9^ Clinicopathological studies have revealed an association between the presence of an RGP and low angiogenic activity,^4^, ^9^, ^10^, ^11^ co‐option of the sinusoidal blood vessels,^4^, ^9^, ^11^ resistance to anti‐angiogenic therapy,^7^ and a poor prognosis.^12^, ^13^, ^14^

Previous reports have indicated that GPs provide useful information for selection of the appropriate treatment and management strategy for malignant tumors. However, reports about the GPs of LMs from PCa are scarce. The reason for this could be that tissue collection from metastatic PCa is usually done by needle biopsy, and not surgical resection, and, therefore, the amount of tumor‐liver interface available in the biopsy specimen is insufficient for assessment of the GP. International consensus guidelines for classification of the histopathological GPs of LMs define an “insufficient tumor‐liver interface” as <20% of the expected interface in tissue sections.^6^ Tissue samples of LMs with a complete circumference are occasionally obtained following an excisional biopsy during diagnostic laparotomy prior to curative‐intent resection for PCa; this allows evaluation of the GPs in PCa.

The objective of this study was to characterize the GPs in LMs in patients with PCa. The circumferential GP at the tumor‐liver interface was first investigated based on international consensus guidelines^6^ in resected LMs. The data revealed that most LMs show a RGP component and a predominantly homogeneous GP. When we identified an RGP component in a portion of a LM from a PCa patient, the LM was considered to be a RGP‐predominant tumor. Therefore, we conducted a clinicopathological study of the RGP using biopsy specimens of LMs obtained from patients with treatment‐naïve advanced PCa.

## 2 MATERIALS AND METHODS

### 2.1 Tissue

The data of resected LMs obtained between 1998 and 2018 from treatment‐naïve PCa patients at our institution were reviewed. Samples that were histopathologically confirmed as metastasis from ductal adenocarcinoma of the pancreas were investigated. A total of 14 resected LMs were available for whole circumferential assessment after analysis of hematoxylin and eosin‐stained sections from formalin‐fixed, paraffin‐embedded (FFPE) samples.

Between 2006 and 2015, 279 liver lesions from distinct 279 treatment‐naïve PCa patients were histopathologically confirmed as being LMs in FFPE percutaneous liver biopsy specimens obtained under continuous real‐time ultrasonographic monitoring using a 21‐ or 18‐gage needle (Sonopsy‐C1; Hakko). The biopsy specimens were checked macroscopically for adequacy. Tissue core specimens were immediately preserved in 10% neutral buffered formalin solution. Hematoxylin and eosin‐stained sections from the FFPE samples were evaluated, and the tumor‐liver interface could be observed in 107 LMs.

### 2.2 Definition of the GP

The GP was determined based on international guidelines for the recognition of GPs.^6^ Briefly, in tumors with the desmoplastic GP, nests of tumor cells were separated from the liver parenchyma by a layer of desmoplastic stroma, with no direct contact between the tumor cells and liver parenchyma. In the pushing type of GP, the liver plate around the LM was compressed and ran parallel to the tumor‐liver interface. In the RGP, the tumor cells and liver parenchyma were in close approximation without compression of the liver cell plates, a desmoplastic stroma, or abundant inflammatory cell infiltration; the tumor cells replaced the hepatocytes in the liver cell plates without destroying the trabecular architecture of the liver. When the histological findings at the periphery of the LMs could not be classified into any typical GP, the GP was labeled as unclassified GP. The sinusoidal GP and portal GP^6^ were not evaluated in this study.

### 2.3 Assessment of the GP

The tumor‐liver interface was evaluated for determining the GP. The relative ratio of each GP that covered a length of >5% of the total length of the interface was recorded. The GP that covered over 50% of the total circumference was defined as the predominant GP. When the relative ratio of coverage of the circumference by a GP was over 80%, that GP was defined as a homogeneous GP. The resected LMs were evaluated by circumferential analysis, while the available tumor‐liver interface was evaluated in the biopsied specimens.

To visualize the precise tumor‐liver interface, the tumor cytoplasm was immunostained with an anti‐cytokeratin 7 monoclonal antibody (clone SP52, dilution ready to use, Roche, Ventana) on the fully automated Ventana Benchmark ULTRA platform (Ventana). Gordon and Sweet's reticulin staining was performed to trace the liver cell plate.

Patients whose specimens were classified RGP‐predominant were classified into the RGP group. Other patients were classified into the non‐RGP group.

### 2.4 Other histological assessments

The following six histological parameters were examined: (a) GP; (b) degree of inflammatory cell infiltration; (c) cellularity of the tumor; (d) predominant differentiation grade of the tumor; (e) presence/absence of tumor necrosis; (f) degree of fibrosis. These factors were assessed at both the periphery and the central area of the tumor. The periphery of the tumor was defined as the portion of the specimen within 200 μm of the tumor‐liver parenchymal interface. The central area of the tumor was defined as the portion of the tumor beyond 200 μm within the tumor‐liver parenchymal interface.

The degree of inflammatory cell infiltration was evaluated at a magnification of 20× at the interface between the LM and liver parenchyma, and was classified as low, intermediate, or severe. Cellularity was defined as the area occupied by the tumor cells per unit area of tumor tissue at a magnification of 20×, and was classified as low (<20%), intermediate (20% to 50%), or high (>50%). The histological differentiation grade of the tumor was graded according to the WHO classification,^15^ and was classified as G1/2 (well/moderately differentiated adenocarcinoma) or G3 (poorly differentiated adenocarcinoma). Tumor necrosis was defined as confluent cell death in invasive areas of the LM that was visible at an objective lens magnification of 4×.^16^ The degree of fibrosis was evaluated at a magnification of 20×.^17^ According to the percent area of fibrosis, intratumoral fibrosis was classified as mild (<20%), intermediate (20% to 50%), or severe (>50%). All slides were evaluated in a blinded manner with respect to the clinical data.

### 2.5 Immunohistochemistry

The primary antibodies and the antigen retrieval methods were described in Table S1. Immunostaining with CD4 and CD8 was performed with the fully automated Ventana Benchmark ULTRA platform (Ventana) according to the manufacturer's instructions.

The microscopic images of CD4, CD8, and FOXP3 immunostaining were obtained using an objective lens of 20× with a NanoZoomer Digital Pathology system (Hamamatsu Photonics). The positive percentages of CD4, CD8, and FOXP3 at the periphery of tumor plus tumor‐liver interface were evaluated. The Automeasure function in Axio Vision 4.9.1 software (Carl Zeiss) was used to distinguish the immunopositive area and to calculate the occupied percentage of the immunopostive cells per tumor at the evaluated area. Positive stain was defined on the basis of the stained signal of the splenocyte as positive control.

### 2.6 Clinical parameters

The age, sex, Eastern Cooperative Oncology Group Performance Status (ECOG‐PS) Scale, primary site, primary tumor size, presence/absence of ascites, long axis of the largest LM, number of LMs, serum level of carbohydrate antigen19‐9 (CA19‐9), serum level of C‐reactive protein (CRP), and the results of the first‐line chemotherapy were obtained from the clinical records. The median values were set as the cutoff for the noncategorical parameters. The patients were divided into the high group (above the median value) or the low group (equal to or below the median value). The cutoff value of the serum CRP was set at 2.0 mg/dL in this study, based on the findings of our previous study.^18^

Overall survival (OS) was defined as the interval between the date of start of chemotherapy and the date of the last follow‐up examination or death. Progression‐free survival (PFS) was defined as the time elapsed between the date of treatment initiation and date of documentation of tumor progression or death from any cause. Tumor progression and the antitumor effect were retrospectively judged based on the new response evaluation criteria in solid tumors as indicated in the revised RECIST guideline version 1.1.^19^

### 2.7 Statistical analysis

The frequencies in the two groups were compared by Fisher's exact test. Comparisons of noncategorical data were performed by the Wilcoxon test. Parameters that were identified as being significantly associated with the PFS or OS were evaluated by univariate analyses using the Cox regression hazard model and then further assessed with multivariate analysis using the Cox regression proportional hazards model. The curves of PFS and OS were drawn by the Kaplan‐Meier method. The significance level was set at *P* < .05, and all *P*‐values were two‐sided. The statistical analyses were performed using the JMP 10 software, Windows version (SAS Institute).

## 3 RESULTS

### 3.1 Circumferential GP analysis

**FIGURE 1:**
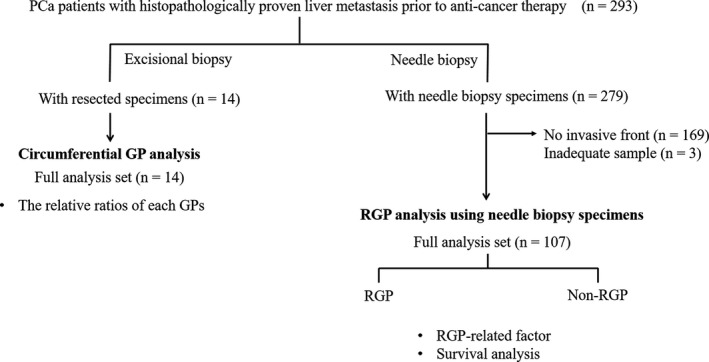
Study flow. A total of 293 PCa patients with pathologically proven liver metastasis prior to the start of anti‐cancer therapy were included in this study. The circumferential GPs were first investigated in 14 LMs resected by excisional biopsy. The data revealed that most LMs had an RGP component and that the majority showed a homogeneous GP. Factors associated with the presence of the RGP and survival analysis were performed using needle biopsy specimens of 107 LMs

A total of 14 lesions from 14 treatment‐naïve PCa patients were resected by excisional biopsy and confirmed as being LMs (median age, 57 years; males, 64%) (Figure 1). Poorly and moderately differentiated adenocarcinomas were diagnosed in 36% and 74% of the LMs, respectively.

**FIGURE 2:**
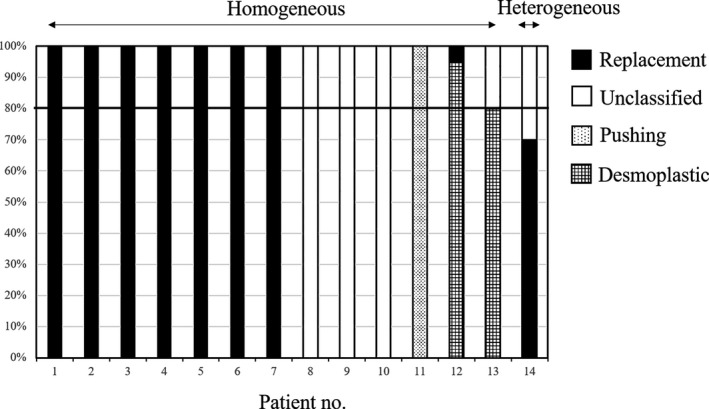
Circumferential analysis using the 14 resected LMs. Thirteen of 14 LMs (93%) showed a homogeneous GP, including the RGP in 7 LMs (50%), desmoplastic GP in 2 LMs (14%), pushing‐type GP in 1 LM (7%), and an unclassified GP in 3 LMs (22%). Predominant‐RGP, desmoplastic GP, pushing‐type GP, and unclassified GP were observed in 8 (57%), 2 (14%), 1 (7%), and 3 (21%) LMs, respectively. Among the 9 LMs showing an RGP component, a predominant RGP was observed in 8 LMs (89%). The GP was regarded as homogeneous if the percentage of the circumference covered by the same GP was over 80%

**FIGURE 3:**
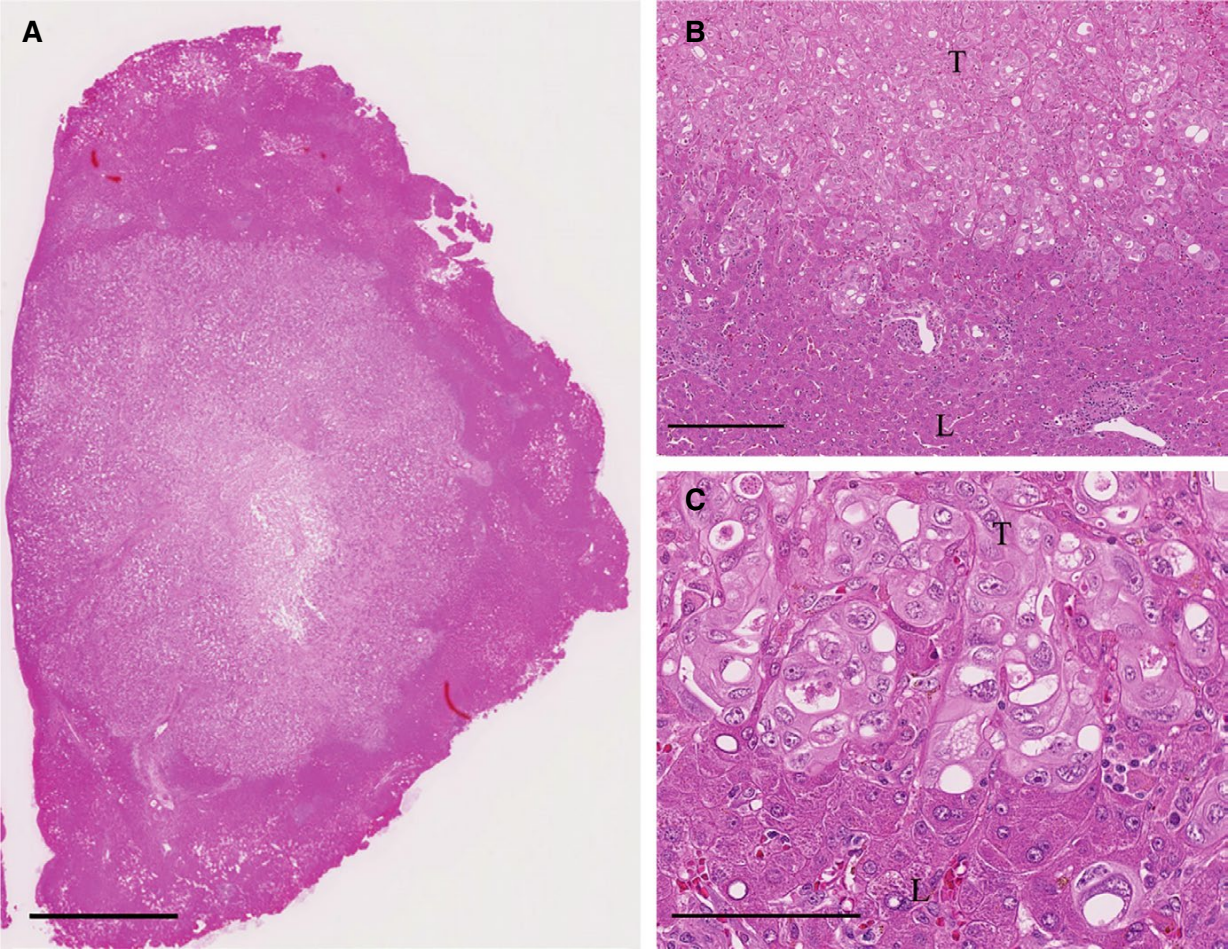
Photomicrographs of a resected liver metastasis showing the replacement GP (hematoxylin and eosin stain). A, Loupe image of the resected liver specimen. Bar, 2500 μm. B, Resected liver specimen containing the tumor periphery. The whole region around the LM shows RGP (original magnification, 10×. Bar, 250 μm). C, RGP, in which the tumor cells replaced the hepatocytes without destroying the trabecular architecture of the liver, with no desmoplastic changes or inflammatory cell infiltration (original magnification, 40×. Bar, 100 μm). T, tumor; L, liver parenchyma

Following circumferential GP analysis (Figure 2), the relative ratios of coverage of the tumor circumference by each GP were recorded for all the 14 LMs (Figure 3). The relative ratio of coverage by the RGP was 100% in 7 LMs, 70% in 1 LM, and 5% in 1 LM. Among the 9 LMs containing an RGP component, the RGP was homogeneous in 7 LMs (78%). The desmoplastic type of GP was observed in 2 LMs, with relative ratios of coverage of 95% and 80%. Pushing‐type and unclassified GP were identified in 1 and 5 LMs, respectively.

Of the 14 LMs 13 (93%) showed a homogeneous GP, including the RGP in 7 LMs (50%), the desmoplastic GP in 2 LMs (14%), the pushing‐type GP in 1 LM (7%), and an unclassified GP in 3 LMs (22%) (Figure 2). Predominance of the RGP, desmoplastic GP, pushing‐type GP, and unclassified GP was observed in 8 (57%), 2 (14%), 1 (7%), and 3 LMs (21%), respectively. A homogeneous or predominant RGP was related to a tendency for a poor prognosis as compared to nonhomogeneous (7.5 vs 16.5 months, *P* = .10) or nonpredominant RGP (8.6 vs 16.5 months, *P* = .20).

In 6 of the 14 patients, the primary tumor was resected, and the patient was diagnosed as having PCa. The fibrotic status of the primary tumor was classified as medullary, intermediate or scirrhous, according to the 7th edition of the General Rules for the Study of Pancreatic Cancer edited by the Japan Pancreas Society. The primary tumors in all 6 patients showed intermediate or scirrhous fibrosis. Half of the LMs in the 6 patients showed the RGP and their fibrotic status was classified as “medullary,” different from that of the primary tumors. Three LMs showed a non‐RGP type of GP and their fibrotic status was classified as “intermediate” or “scirrhous”.

### 3.2 RGP analysis using needle biopsy samples

A total of 107 biopsy samples of LMs obtained from 107 treatment‐naïve PCa patients were examined (Figure 1). The clinical data of the 107 PCa patients from whom biopsy specimens of the LMs were obtained before the start of chemotherapy are summarized in Table 1.

**TABLE 1:** Baseline characteristics

Parameter	Median (IQR)	All, N (%)	Growth pattern		*P*‐value
			Replacement, N (%)	Non replacement, N (%)	
		107 (100)	54 (100)	53 (100)	
Age (y)	64 (59‐71)				
>65		51 (48)	25 (46)	26 (49)	.84
≤65		56 (52)	29 (54)	27 (51)	
Gender					
Male		71 (66)	38 (70)	33 (62)	.42
Female		36 (34)	16 (30)	20 (38)	
PS					
0		52 (49)	24 (44)	31 (58)	.18
≥1		55 (51)	30 (56)	22 (41)	
Primary location					
Head		22 (21)	13 (24)	9 (17)	.48
Primary size (mm)	44 (35‐36)				
>Median		55 (51)	27 (50)	28 (53)	.85
Ascites					
Present		28 (79)	17 (31)	11 (21)	.27
Size of liver metastasis (mm)	25 (18‐35)				
>Median		50 (47)	26 (48)	24 (45)	.85
Number of liver metastases	20 (7‐40)				
>Median		49 (46)	32 (59)	17 (32)	<.01
CA19‐9 (U/mL)	4111 (444‐32 000)				
>Median		53 (50)	29 (54)	24 (45)	.44
CRP (mg/dL)	1.08 (0.29‐2.6)				
>Median		56 (52)	31 (57)	25 (47)	.34
≥2		39 (36)	24 (44)	15 (28)	.11
Chemotherapy					
GEM monotherapy		42 (39)	19 (35)	23 (43)	.25
S‐1 monotherapy		6 (6)	2 (4)	4 (8)	
GEM + S‐1		4 (4)	4 (7)	0 (0)	
GEM + Erlotinib		18 (17)	10 (19)	8 (15)	
GnP		9 (8)	3 (6)	6 (11)	
GEM + Investigational agent		11 (10)	5 (9)	6 (11)	
FOLFIRINOX		17 (16)	11 (20)	6 (12)	

Note

Pearson's chi‐square test or Fisher's exact test was used to compare qualitative variables. The significance level was set at *P* < .05.

Abbreviations: CA19‐9, carbohydrate antigen19‐9; CRP, C‐reactive protein; FOLFIRINOX, triplet regimen that contains oxaliplatin, irinotecan, fluorouracil, and leucovorin; GEM, Gemcitabine; GnP, GEM + nab Paclitaxel; IQR, interquartile range; PS, performance status.

**FIGURE 4:**
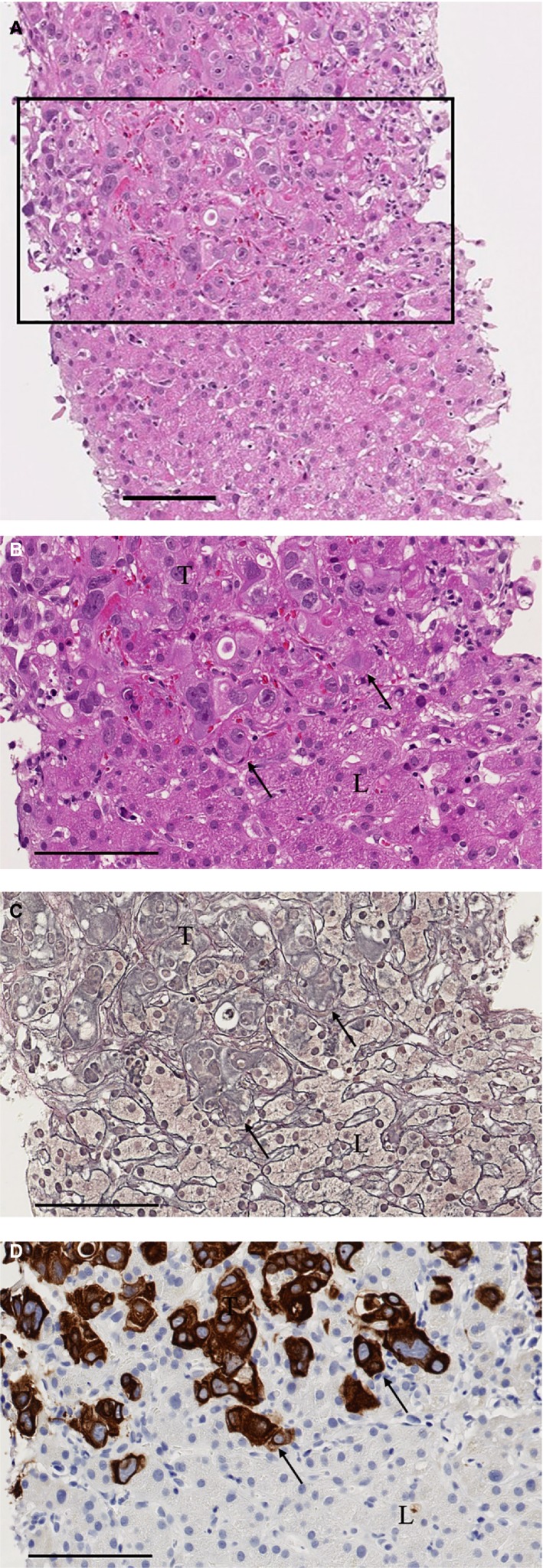
Photomicrographs of biopsied liver metastasis showing the RGP (hematoxylin and eosin staining, Gordon‐Sweet's reticulin staining, and immunostaining). RGP is represented in (A) and (B) with hematoxylin and eosin staining (original magnification, (A) 10×, (B) 40×. Bar, 100 μm), in (C) with Gordon‐Sweet's silver staining (original magnification, 40×. Bar, 100 μm), and in (D) with cytokeratin 7 staining (original magnification, 40×. Bar, 100 μm). The region in the black box in (A) is shown in (B). The RGP shows no desmoplastic stroma or inflammatory cells at the invasive front (black arrows). In (A) and (B), the tumor periphery is difficult to discern, but in (C) and (D), Gordon‐Sweet's silver staining was helpful for the detection of sinusoids and evaluation of preservation of the liver cell plate in the tumor. Cytokeratin 7 staining clearly revealed that the tumor cells and liver parenchyma were in close approximation, without compression of the liver cell plates. T, tumor; L, liver parenchyma

An image of a typical replacement GP is shown in Figure 4. Gordon‐Sweet's silver staining and cytokeratin 7 staining clearly showed that the tumor cells and liver parenchyma were in close approximation, without compression of the liver cell plates. The RGP was observed in 50% of the 107 specimens (n = 54; Table 2), and these PCa patients were classified into the RGP group. The remaining 53 patients, including those with the desmoplastic GP (n = 22, 21%) and unclassified GP (n = 31, 29%), were classified into the non‐RGP group. There was no case of the pushing type of GP. Images of a desmoplastic and unclassified GP were shown in Figure S1.

**TABLE 2:** Morphological features of the replacement and non‐replacement growth pattern of liver metastasis

Parameter	Replacement, N (%)	Non‐replacement, N (%)	*P*‐value
All	54 (100)	53 (100)	
Predominant replacement	54 (100)	0 (0)	
Predominant desmoplastic	0 (0)	31 (58)	
Predominant pushing	0 (0)	0 (0)	
Predominant unclassified	0 (0)	22 (42)	
Inflammatory cell infiltration at the interface between LM and liver parenchyma			
Low	42 (78)	27 (51)	<.01
Intermediate/High	12 (22)	26 (49)	
Cellularity at the periphery of the tumor			
Low	5 (9)	26 (49)	<.01
Intermediate/High	49 (91)	27 (51)	
Cellularity at the central area of the tumor			
Low	37 (68)	45 (85)	.04
Intermediate/High	17 (32)	8 (15)	
Differentiation at the periphery of the tumor			
Well/mod	1 (2)	19 (36)	<.01
Poor	53 (98)	34 (64)	
Differentiation at the central area of the tumor			
Well/mod	30 (56)	34 (64)	.36
Poor	24 (44)	19 (36)	
Necrosis			
Present	20 (37)	11 (21)	.06
Absent	34 (63)	42 (79)	
Fibrosis at the periphery of the tumor			
Mild	48 (89)	26 (49)	<.01
Intermediate/Severe	6 (11)	27 (51)	
Fibrosis at the central area of the tumor			
Mild	20 (37)	10 (19)	.04
Intermediate/Severe	34 (63)	43 (61)	

Note

Well; well differentiated, Mod; moderately differentiated, Poor; poorly differentiated. Predominant differentiation in each patient was defined as the most frequently observed type of differentiation.

Pearson's chi‐square test or Fisher's exact test was used to compare qualitative variables. The significance level was set at *P* < .05.

The histological features of the LMs in the RGP group were characterized by minimal inflammatory cell infiltration at the interface between the LM and the liver parenchyma (78% in the RGP group vs 51% in the non‐RGP group, *P* < .01), high cellularity at the periphery of the tumor (91% vs 51%, *P* = .04), high cellularity in the central area of the tumor (32% vs 15%, *P* = .04), poor tumor differentiation at the periphery of the tumor (98% vs 64%, *P* < .01), and mild fibrosis in both the peripheral (89% vs 49%, *P* < .01) and central areas of the tumor (37% vs 19%, *P* = .04). A clinical feature of the RGP group was that the mean number of LMs in this group appeared to be higher as compared to that in the non‐RGP group (*P* < .01, Table 1). Univariate analysis identified inflammatory cell infiltration at the interface between LM and liver parenchyma low (*P* < .01), cellularity at the periphery of tumor high (*P* = .03), cellularity at the central area of tumor high (*P* = .01), fibrosis at the periphery of tumor low (*P* = .02), and fibrosis at the central area of tumor low (*P* = .03) as pathological factors associated with a poor prognosis. Multivariate analysis identified inflammatory cell infiltration at the interface between LM and liver parenchyma low (HR: 1.89, 95% CI: 1.17‐3.15 *P* < .01) as the pathological factors that were independently associated with a poor prognosis (Table 3).

**TABLE 3:** Univariate and multivariate analysis of independent significant pathological factors associated with overall survival

Varidated facter	Univariate			Multivariate		
	HR	95% CI	*P*‐value	HR	95% CI	*P*‐value
Inflammatory cell infiltration at the interface between LM and liver parenchyma						
Intermediate/High (reference)	1.00					
Low	1.95	0.55‐3.12	<.01	1.89	1.17‐3.15	<.01
Cellularity at the periphery of tumor						
Low/Intermediate (reference)	1.00					
High	1.68	1.04‐2.82	.03	1.03	0.59‐1.87	.91
Cellularity at the central area of tumor						
Low/Intermediate (reference)	1.00					
High	1.92	1.16‐3.09	.01	1.36	0.68‐2.65	.38
Differentiation at the periphery of tumor						
Well/mod (reference)	1.00					
Por	1.75	0.99‐3.39	.06			
Differentiation at the central area of tumor						
Well/mod (reference)	1.00					
Por	1.53	0.98‐2.36	.06			
Necrosis						
Abscent (referece)	1.00					
Present	1.38	0.87‐2.14	.16			
Fibrosis at the periphery of tumor						
Intermediate/High (reference)	1.00					
Low	1.72	1.09‐2.81	.02	1.51	0.90‐2.60	.12
Fibrosis at the central area of tumor						
Intermediate/High (reference)	1.00					
Low	1.70	1.04‐2.69	.03	1.43	0.72‐2.08	.30

**FIGURE 5:**
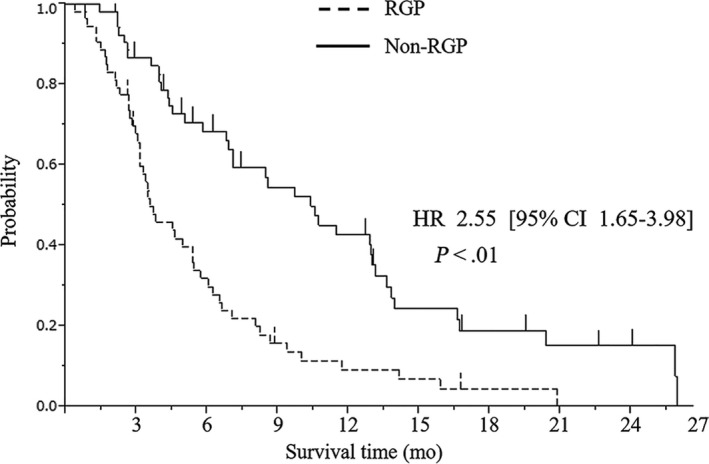
Overall survival (OS) curves of PCa patients with liver metastasis based on RGP analysis using needle biopsy specimens. CI, confidence interval; HR, hazard ratio

The OS, PFS, and tumor response to chemotherapy were analyzed in the 107 patients from whom needle biopsy specimens of the LMs were obtained. The median OS times in all 107 patients, the RGP group, and the non‐RGP group were 6.0 months (95% confidence interval [CI]: 4.5‐7.1), 3.6 months (95% CI: 3.2‐5.4), and 10.4 months (95% CI: 6.9‐13.0), respectively (Figure 5). According to first line chemotherapy, the patients were departed into two groups, GEM+nabPTX (GnP)/FOLFIRINOX and Others. The median OS times in the GnP/FOLFIRINOX and the Others were 8.2 vs 5.4 months (HR: 0.62, 95% CI: 0.39‐1.25, *P* = .07), respectively. In the GnP/FOLFIRINOX, the median OS times in the RGP group and the non‐RGP group were 4.5 vs 13.0 months (HR: 3.6, 95% CI: 1.29‐14.2, *P* = .01), respectively. In the Others, the median OS times in the RGP group and the non‐RGP group were 3.5 vs 8.6 months (HR: 2.5, 95% CI: 1.52‐3.99, *P* < .01), respectively (Table S2).

Univariate analysis identified ECOG‐PS ≥1 (*P* < .01), a high number of LMs (*P* = .01), elevated serum CA19‐9 (*P* = .04), elevated serum CRP (*P* < .01), and RGP (*P* < .01) as factors associated with a poor prognosis. Multivariate analysis identified elevated serum CRP (HR: 2.87, 95% CI: 1.73‐4.75 *P* < .01) and RGP (HR: 2.18, 95% CI: 1.39‐3.46 *P* < .01) as the only two of the above factors that were independently associated with a poor prognosis (Table 4).

**TABLE 4:** Univariate and multivariate analysis of independent significant factors associated with overall survival

Varidated factor	Univariate			Multivariate		
	HR	95% CI	*P*‐value	HR	95% CI	*P*‐value
Age (y)						
<65 (reference)	1.00					
≧65	0.84	0.55‐1.29	.43			
Sex						
Male (reference)	1.00					
Female	0.97	0.60‐1.51	.89			
PS						
0 (reference)	1.00					
≧1	1.70	1.16‐2.51	<.01	1.5	0.97‐2.30	.07
Primary location						
Body and tail (reference)	1.00					
Head	1.30	0.75‐2.14	.33			
Primary size (mm)						
≦44 (reference)	1.00					
>44	1.48	0.96‐2.30	.07			
Ascites						
Abscent (referece)	1.00					
Present	1.50	0.93‐2.37	.09			
Size of liver metastasis (mm)						
≦25 (reference)	1.00					
>25	1.39	0.90‐2.12	.13			
Number of liver metastasis						
≦20 (referece)	1.00					
>20	1.74	1.14‐2.69	.01	1.09	0.67‐1.78	.74
CA19‐9 (U/mL)						
≦4111 (reference)	1.00					
>4111	1.56	1.02‐2.39	.04	1.25	0.79‐2.01	.33
CRP (mg/dL)						
<2 (reference)	1.00					
≧2	3.31	2.10‐5.18	<.01	2.73	1.62‐4.63	<.01
Growth pattern						
Non‐replacement (reference)	1.00					
Replacement	2.56	1.65‐3.98	<.01	2.27	1.33‐3.38	<.01
Chemotherapy rejimen						
Others (refrence)	1.00					
GnP/FOLFIRINOX	0.62	0.34‐1.04	.07			
Inflammatory cell infiltration at the interface between LM and liver parenchyma						
High/Intermediate (reference)	1.00					
Low	1.95	0.55‐3.12	<.01	1.17	0.70‐1.97	.55

Note

Others in chemotherapy rejimens contain GEM monotherapy, S1 monotherapy, GEM + S1, GEM + Erlotinib and GEM + Investigating agent.

Abbreviations: CI, confidence interval; HR, hazard ratio.

The median PFS in the 107 patients was 2.8 months (95% CI: 1.9‐3.4), that in the RGP group was 1.5 months (95% CI: 1.3‐2.3), and that in the non‐ RGP group was 3.6 months (95% CI: 3.2‐5.5).

The response to chemotherapy was evaluated in the 107 patients. There were no cases of complete response (CR). A partial response (PR) and stable disease (SD) were observed in 22 (21%) and 37 patients (35%), respectively. The overall response rate (ORR) was defined as the percentage of patients in whom CR or PR was achieved. The disease control rate (DCR) was defined as the percentage of patients in whom CR, PR, or SD was achieved. The DCR in the RGP group was lower than that in the non‐RGP group (37% vs 74%, *P* < .01). The ORR in the RGP group tended to be lower than that in the non‐RGP group (15% vs 26%, *P* = .15).

### 3.3 Immunohistochemistry for CD4, CD8, and FOXP3

In order to investigate the distribution and prognostic impacts of T lymphocytes in LM, immunohistochemical analysis was performed using anti‐CD4, CD8, and FOXP3 antibody in 98 patients of this study. The positive percentage of CD4, CD8, and FOXP3 at the periphery of LM and tumor‐liver interface were 2.29, 1.08, and 0.09% in median, respectively. Median value of CD8 positive percentage in RGP was lower than that in non‐RGP (0.75 vs 1.46, *P* = .04; Figure S2). There were not significant differences of CD4 (1.76 vs 2.84 in median, *P* = .16) and FOXP3 positive percentage (0.08 vs 0.09, *P* = .54) between RGP and non‐RGP. According to median values of positive percentages, the patients were divided into high and low group. Median OS times in low CD4 and CD8 groups were 3.7 months (95% CI: 2.7‐7.0) and 4.5 months (95% CI: 3.2‐6.2), respectively, and tend to be shorter than those in high CD4 (6.8 months, 95% CI: 4.5‐10.0, *P* = .16) and CD8 (8.2 months, 95% CI: 4.0‐10.4, *P* = .37; Figure S2). FOXP3 positive percentage was not associated with OS (low vs high, 5.1 vs 6.0 months, *P* = .29).

## 4 DISCUSSION

The circumferential analysis revealed that the histological GP was homogeneous at the invasive front in the majority of LMs in the patients with PCa. While, according to one report, a ‘‘pure’’ GP (100%) was observed in one‐third of LMs in cases of colorectal carcinoma,^6^ the rate of “pure” GP in the LMs in the patients with PCa in this study was higher, at 79%. A homogeneous GP was defined as a relative coverage rate of the circumference by a GP of >80% in this study, and was observed in 93% of the resected LMs. When the LMs from PCa showing a RGP component, the mean relative coverage rate to the total length by the RGP was 86%. A homogeneous RGP was observed in 7 of the 9 LMs with RGP. These data suggest that the RGP was the representative type of GP in LMs from PCa. The morphological features of metastatic tumors are affected by the background of the metastatic tissue, such as organotropism.^20^ When hepatic organotropism resulted in the RGP of the tumor in patients with PCa, a lower correlation was present between the fibrotic status of the primary tumor and that of the LM. In this study, half of the primary tumors had converted into minimally fibrotic tumors in the hepatic tissue, ie, exhibited a RGP. The tumor‐liver interaction may influence the morphological aspects of RGP in LMs from PCa.

Replacement growth pattern was identified as an important poor prognostic factor in patients with PCa, even after adjustment for elevated serum CRP (≥2.0 mg/dL). Numerous studies have identified elevated serum CRP as a strong predictor of a poor prognosis in treatment‐naïve advanced PCa patients.^2^, ^18^, ^21^ A prognostic factor that is independent of the serum CRP would be expected to have a very strong impact on the prognosis. Thus, the RGP in LMs is a poor prognostic factor not only in cases of colorectal carcinoma, breast adenocarcinoma and urothelial carcinoma, but also in cases of PCa.

Minimal inflammation and mild fibrosis were identified as morphological features of the RGP in PCa. Low grades of fibrosis and inflammatory cell infiltration were observed in 89% and 78% of the biopsy specimens of the LMs in the RGP group, and these rates were much higher as compared to the corresponding rates in the non‐RGP group. These morphological features of the LMs in the RGP group in PCa were comparable to those reported previously for such metastases in patients with other types of cancer.^4^, ^9^ Desmoplastic reaction is thought to represent a host defense mechanism.^22^, ^23^ Two groups have reported, based on studies in mouse models of experimental pancreatic tumors, that depletion of the tumor stroma is associated with tumor progression, a poor prognosis, and resistance to antitumor therapy.^24^, ^25^ In this study, the DCR and ORR in the RGP group were lower than those in the non‐RGP group (37% vs 74%, *P* < .01, and 15% vs 26%, *P* = .15, respectively), which indicated that the RGP conferred resistance to antitumor therapy.

The average number of LMs per patient was higher in the RGP group than in the non‐RGP group in this study. We speculate that the vessel co‐option system could be a cause of dissemination of tumor cells in the liver; LMs showing the RGP can become vascularized with the pre‐existing sinusoidal vessels.^4^, ^9^, ^11^, ^26^ Tumor cells in the RGP group showed invasion along the sinusoidal vessels, suggesting that the tumor cells may spread via sinusoidal vessel flow. Dissemination via vessel co‐option of the sinusoidal flow may have contributed to the larger number of LMs in the RGP group.

The limitations of this study were that the patients included in the survival analysis were receiving multiple regimens of chemotherapy and the deviation of concordance between the results of circumferential and the biopsy‐sample analysis of the GP. The imbalance in the distribution of the chemotherapeutic regimens may have affected the results of the survival analysis. Several chemotherapeutic regimens were used in the patients enrolled in this study. However, we found no significant differences in the distribution of the regimens between the RGP and non‐RGP groups (Table 1). Thus, the potential bias arising from the use of multiple regimens was considered to be very low in this study. Agreement of the biomarker status between needle biopsy specimens and the subsequent surgical specimens has been reported in cases of breast cancer.^27^, ^28^ To overcome limitations in relation to heterogeneity in LMs from PCa, further study is needed to elucidate the concordance rate of the results of assessment of the GP between circumferential and the biopsy‐sample analysis.

This was the first study conducted to evaluate the prognostic significance of the GP observed in the LMs of patients with PCa. The RGP was observed in 50% of the LMs in the PCa patients enrolled in this study, and was identified as an important determinant of a poor prognosis. Identification of the RGP in LMs is expected to be useful as a determinant of the prognosis and for clarifying the tumor biology in PCa patients.

## CONFLICTS OF INTEREST

The authors have no conflicts of interest to declare.

## AUTHORS’ CONTRIBUTIONS

KW designed the study, and wrote the initial draft of the manuscript. SM contributed to the analysis and interpretation of data, and assisted in the preparation of the manuscript. All other authors have contributed to data collection and interpretation, and critically reviewed the manuscript. All authors approved the final version of the manuscript, and agreed to be accountable for all aspects of the work in ensuring that questions related to the accuracy or integrity of any part of the work are appropriately investigated and resolved.

## ETHICS APPROVAL AND CONSENT TO PARTICIPATE

The study protocol was reviewed and approved by the institutional review boards of the National Cancer Center Hospital East. This study was conducted in accordance with the Declaration of Helsinki.

## CONSENT FOR PUBLICATION

Written informed consent was obtained from the study participants, including consent to participate and to publish the findings.

## Supporting information

FigS1A

FigS1B

FigS1C

FigS1D

FigS2A

FigS2B

FigS2C

FigS2D

FigS2E

FigS2F

TableS1‐S2

figure legends

## Data Availability

The data that support the findings of this study are available from the corresponding author upon reasonable request.
