# Supplementary figures and images for: The “histological replacement growth pattern” represents aggressive invasive behavior in liver metastasis from pancreatic cancer
Source: Cancer Med. 2020 Mar 5;9(9):3130–41. doi: 10.1002/cam4.2954 (PMC7196051; doi:10.1002/cam4.2954)

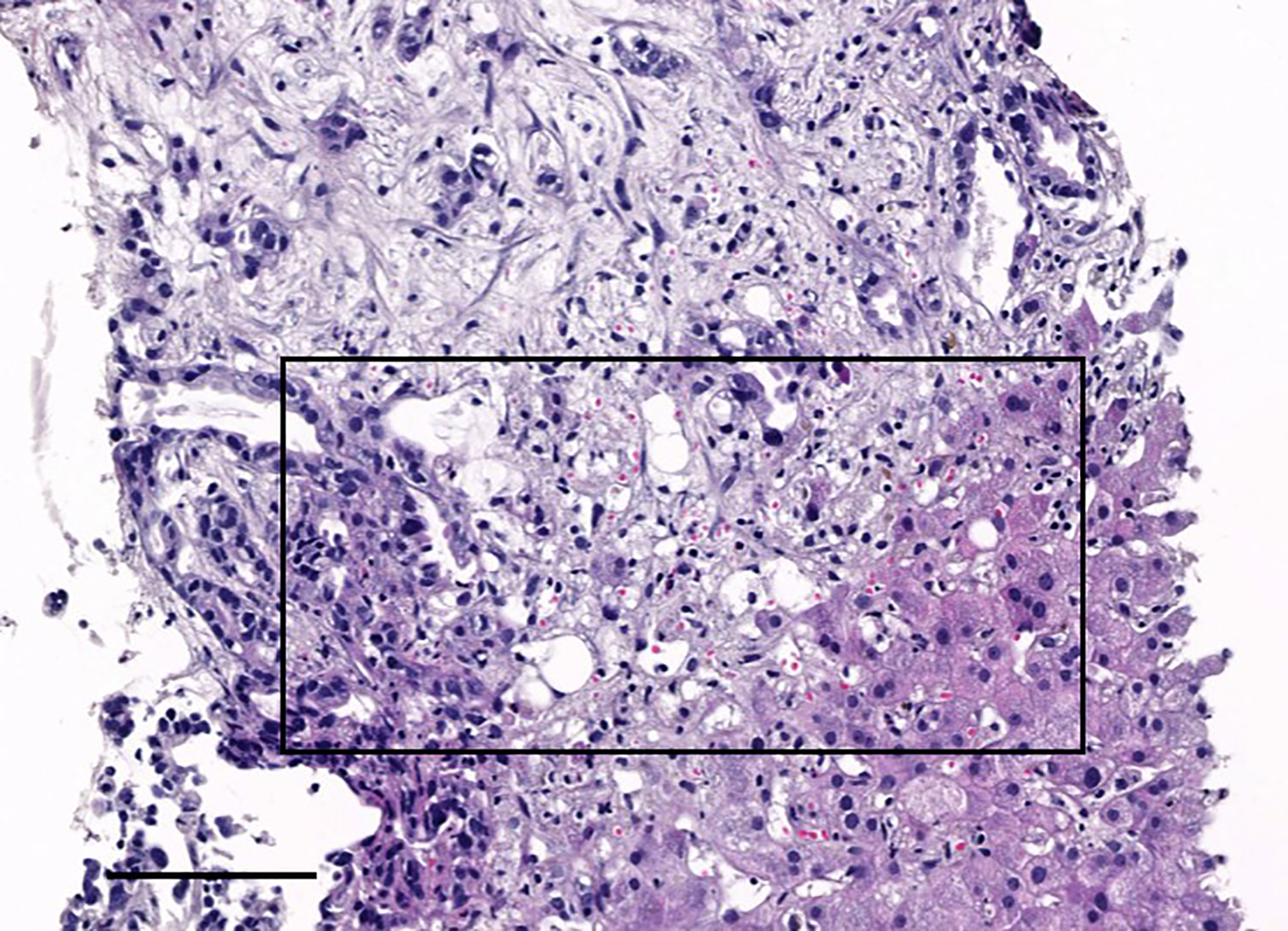

Supplement: Supplementary file 1 — FigS1A [file CAM4-9-3130-s001.tif]

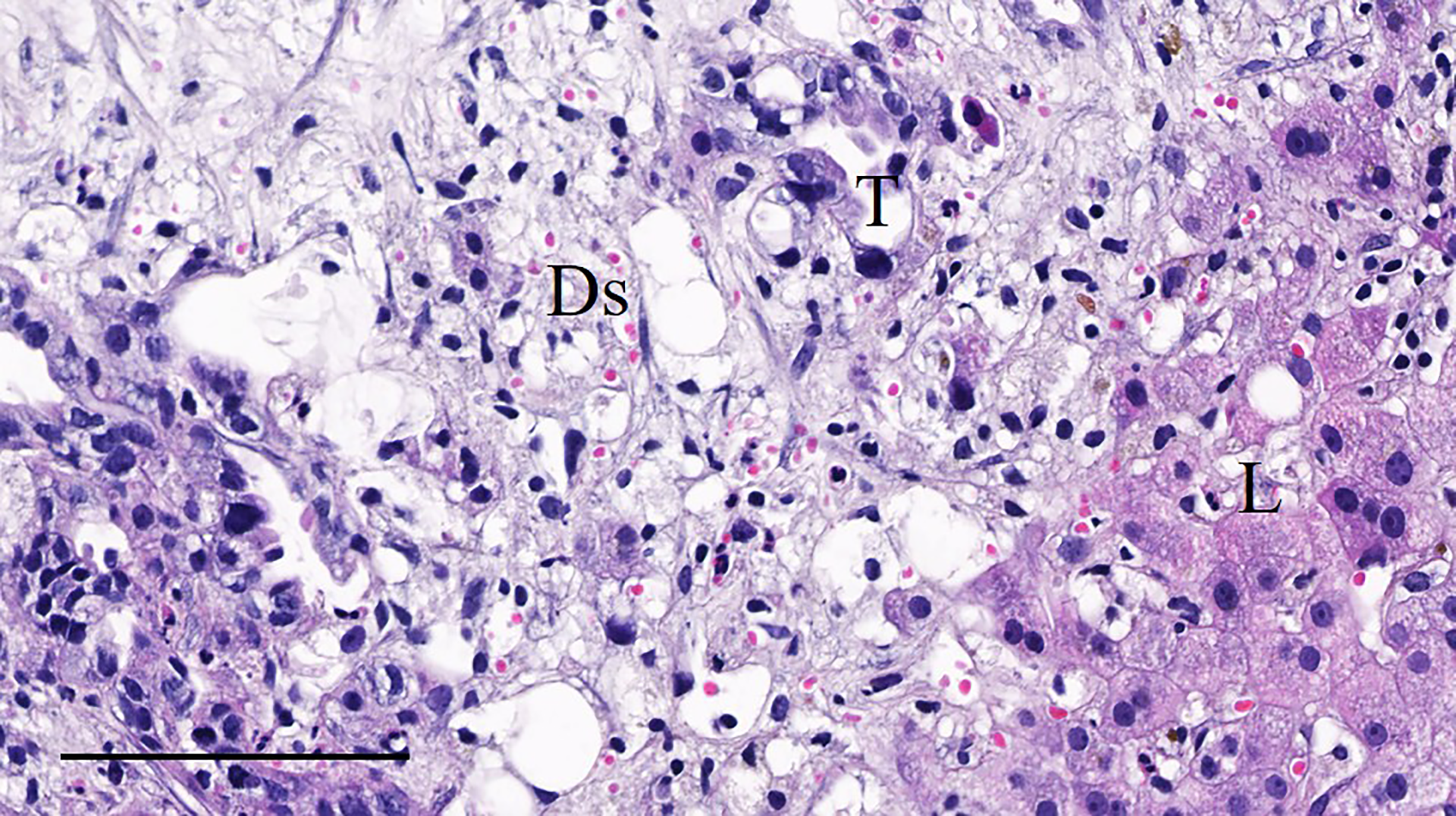

Supplement: Supplementary file 2 — FigS1B [file CAM4-9-3130-s002.tif]

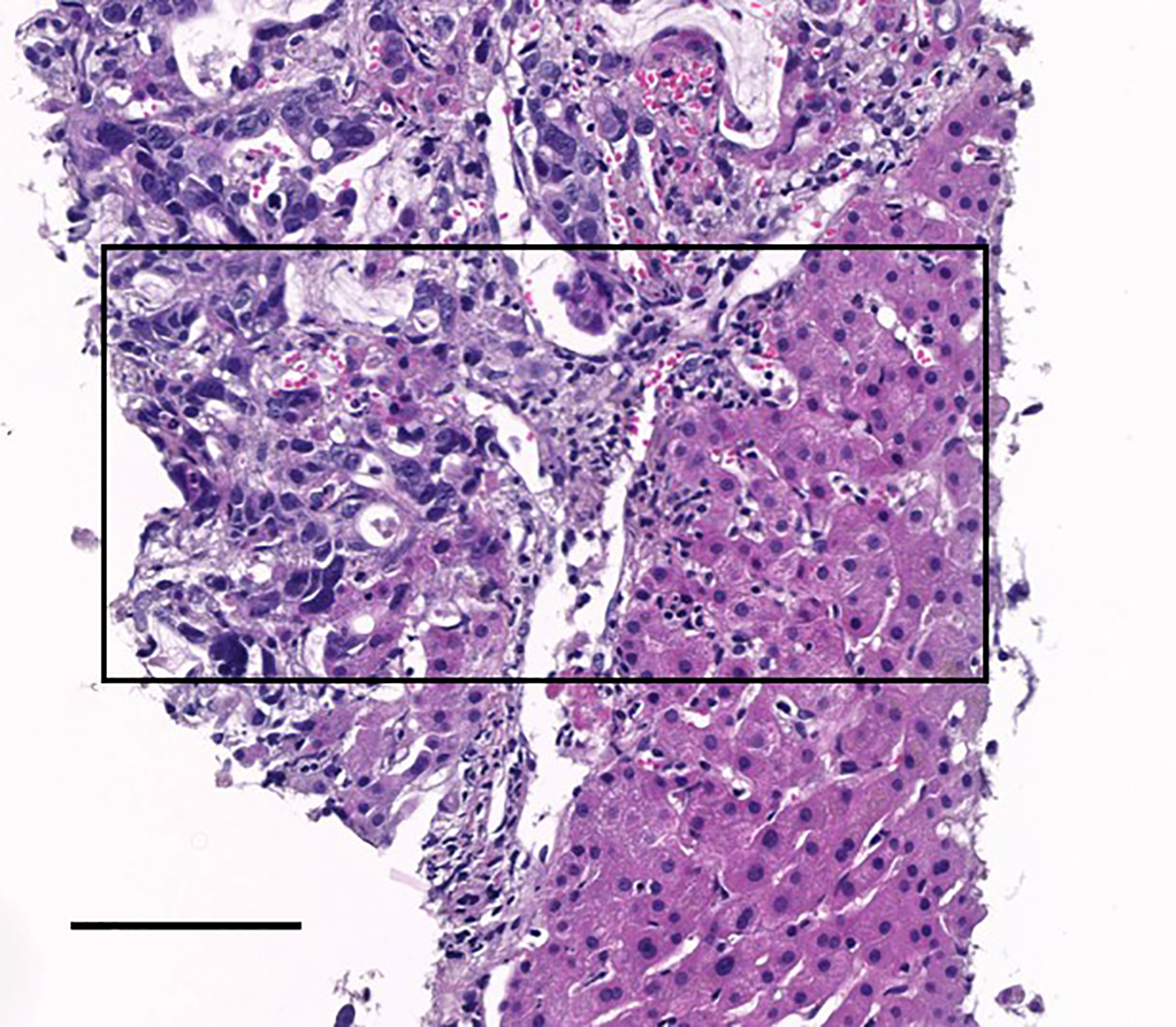

Supplement: Supplementary file 3 — FigS1C [file CAM4-9-3130-s003.tif]

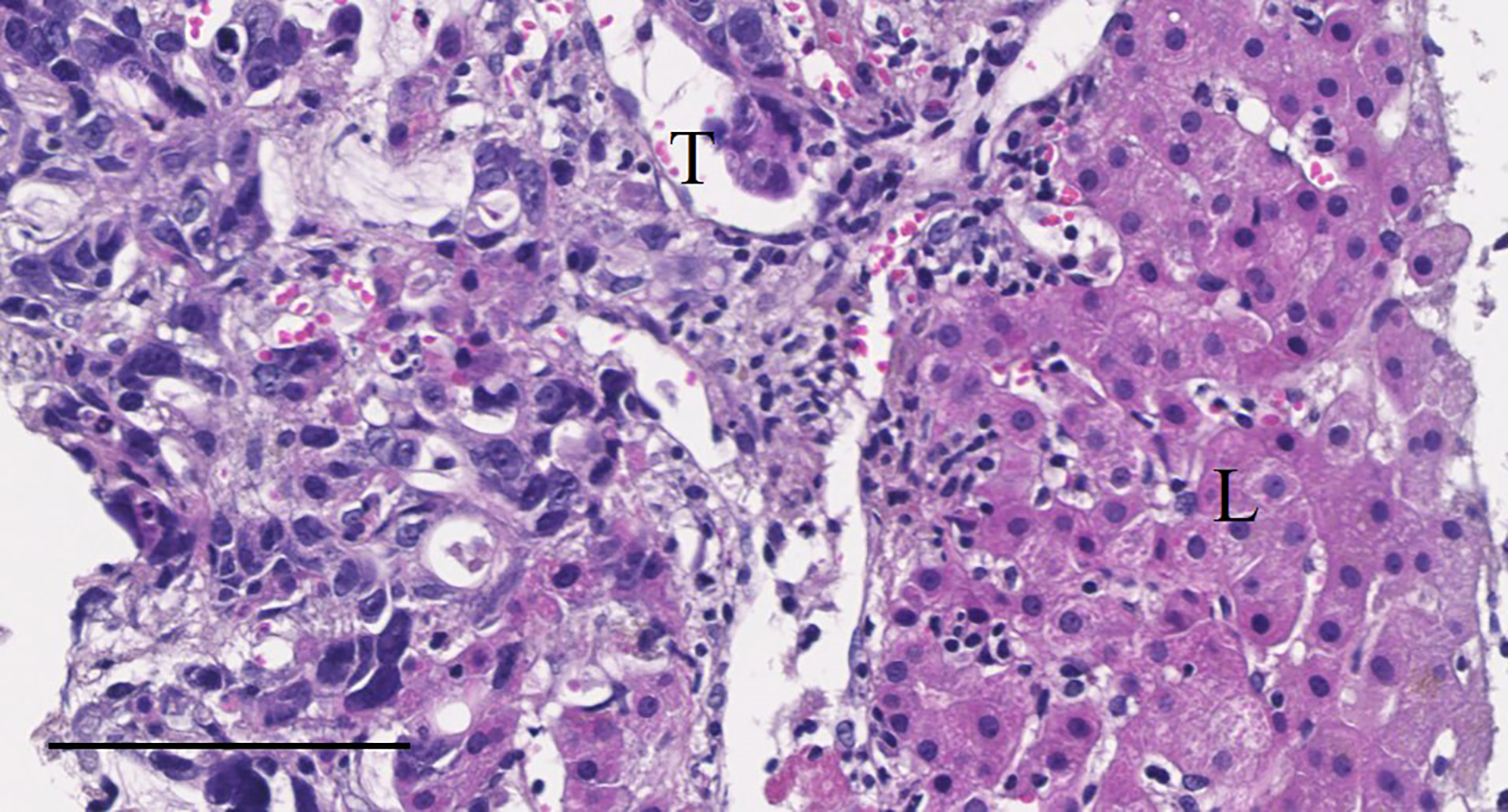

Supplement: Supplementary file 4 — FigS1D [file CAM4-9-3130-s004.tif]

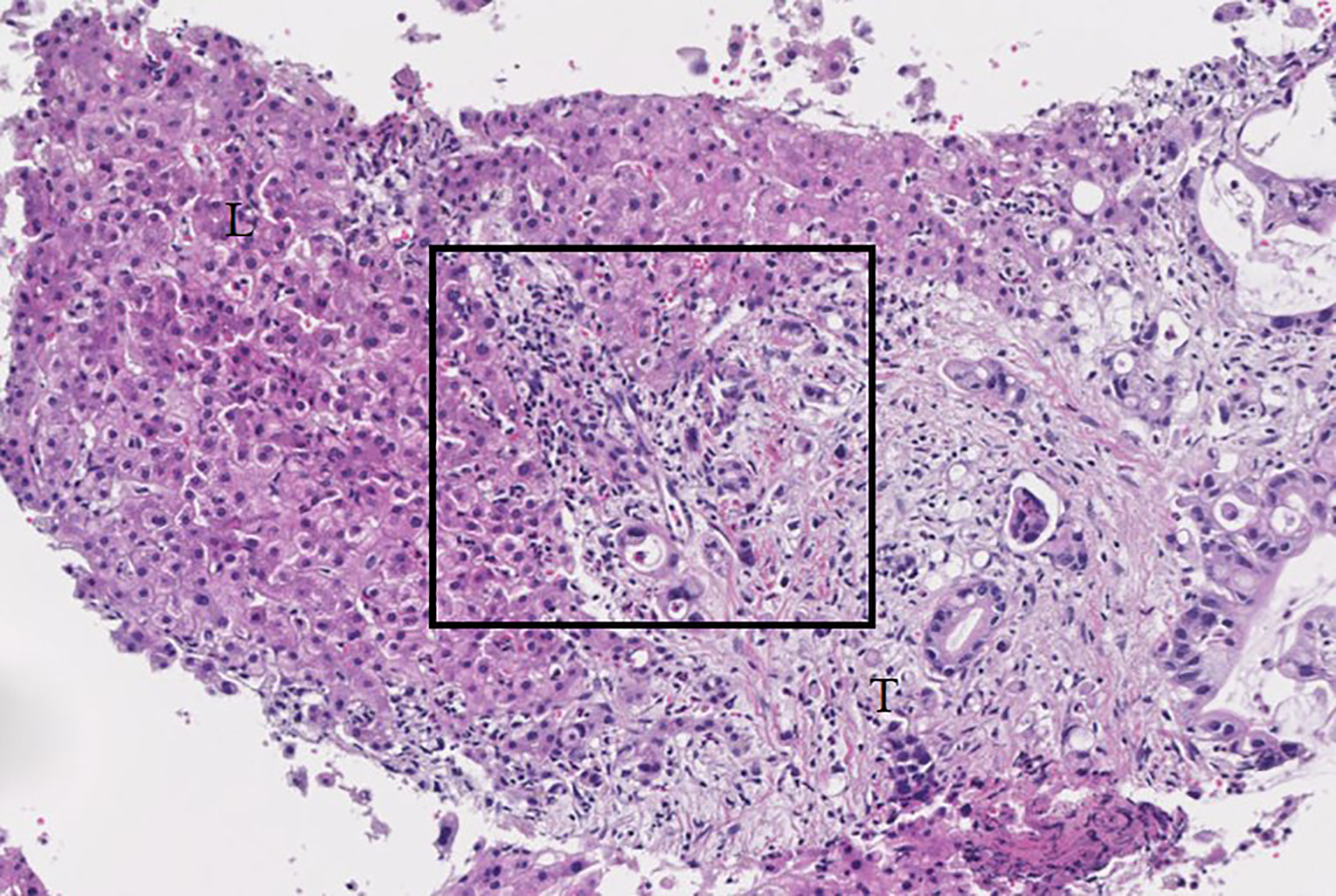

Supplement: Supplementary file 5 — FigS2A [file CAM4-9-3130-s005.tif]

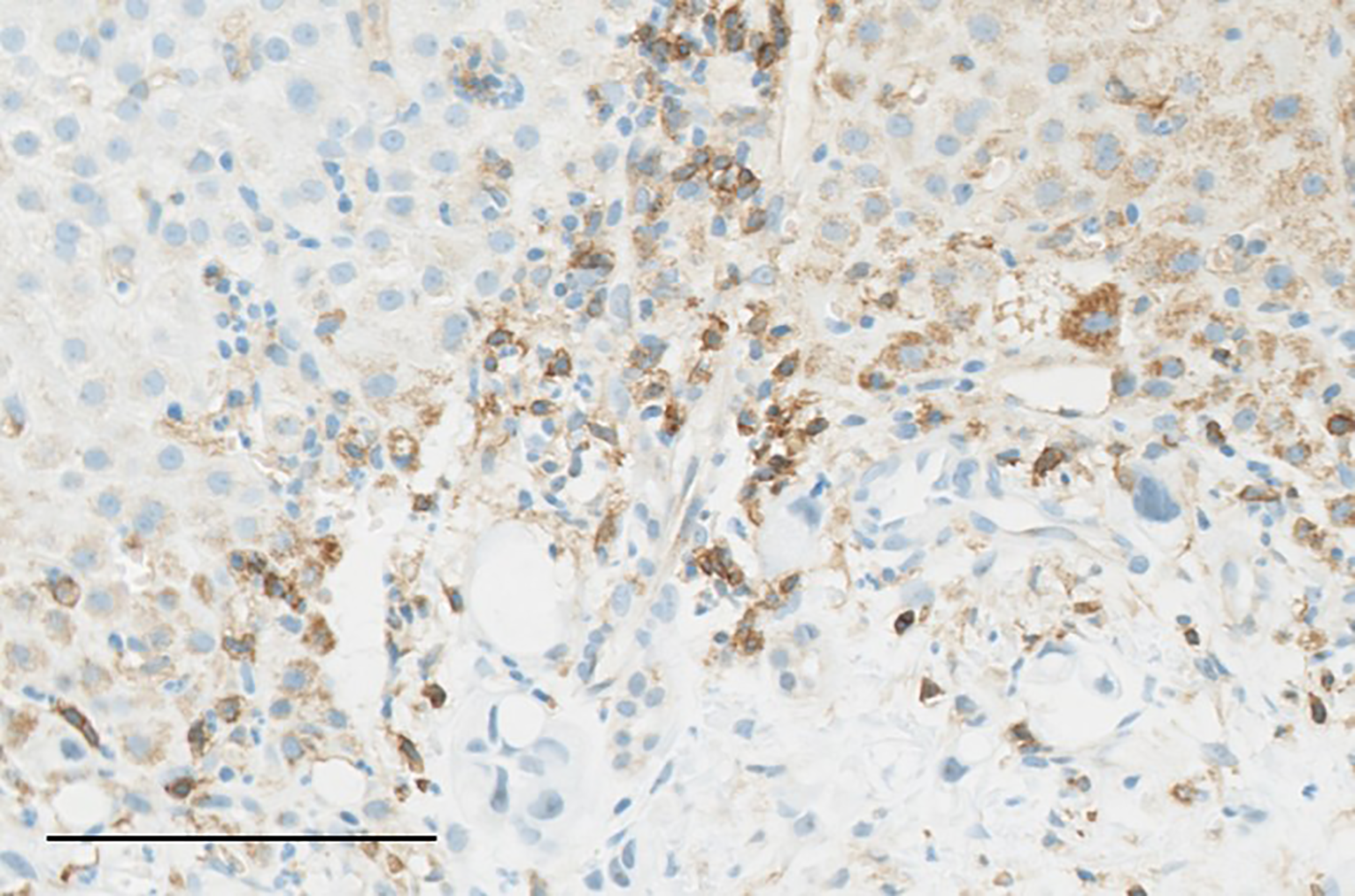

Supplement: Supplementary file 6 — FigS2B [file CAM4-9-3130-s006.tif]

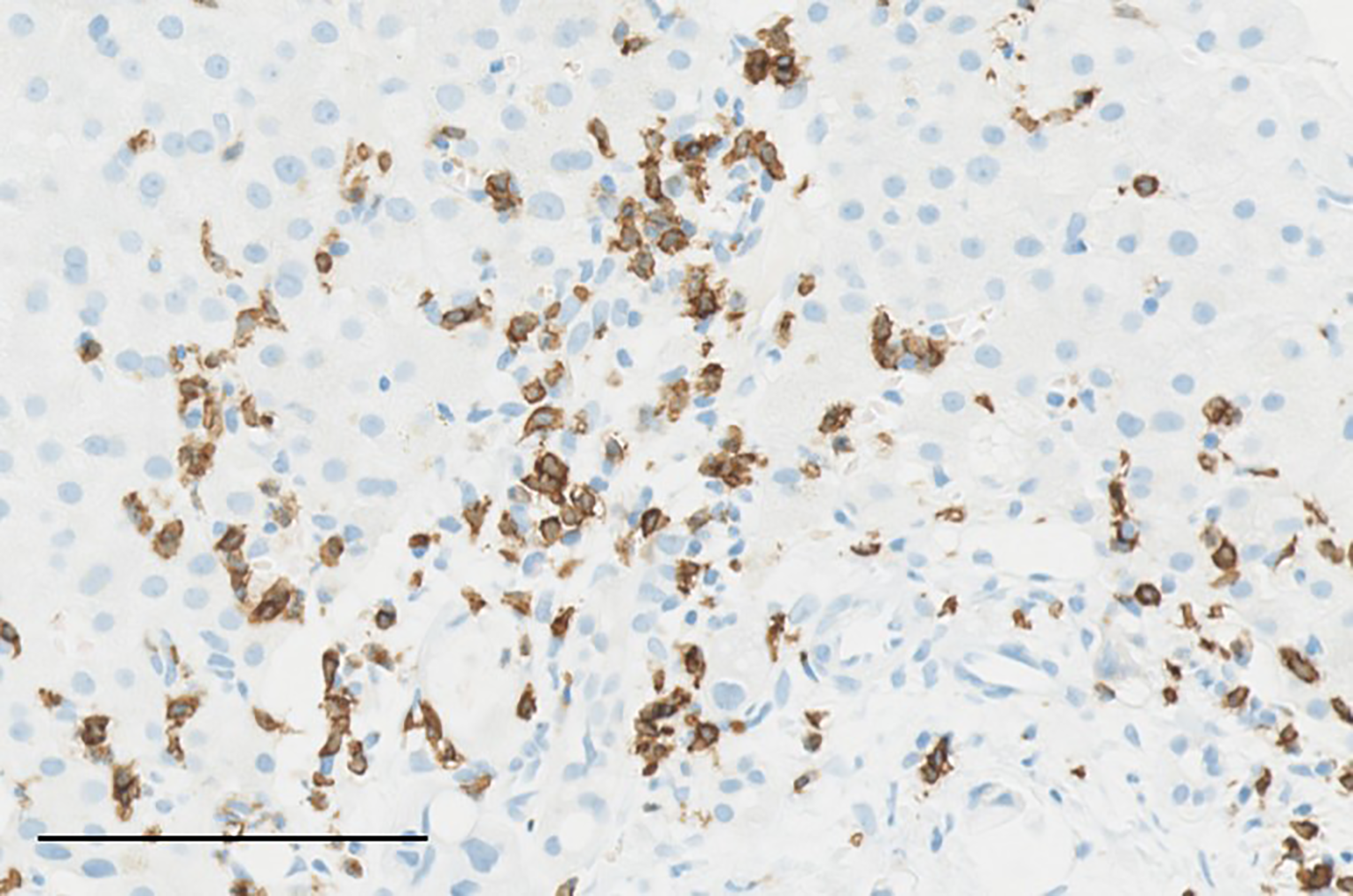

Supplement: Supplementary file 7 — FigS2C [file CAM4-9-3130-s007.tif]

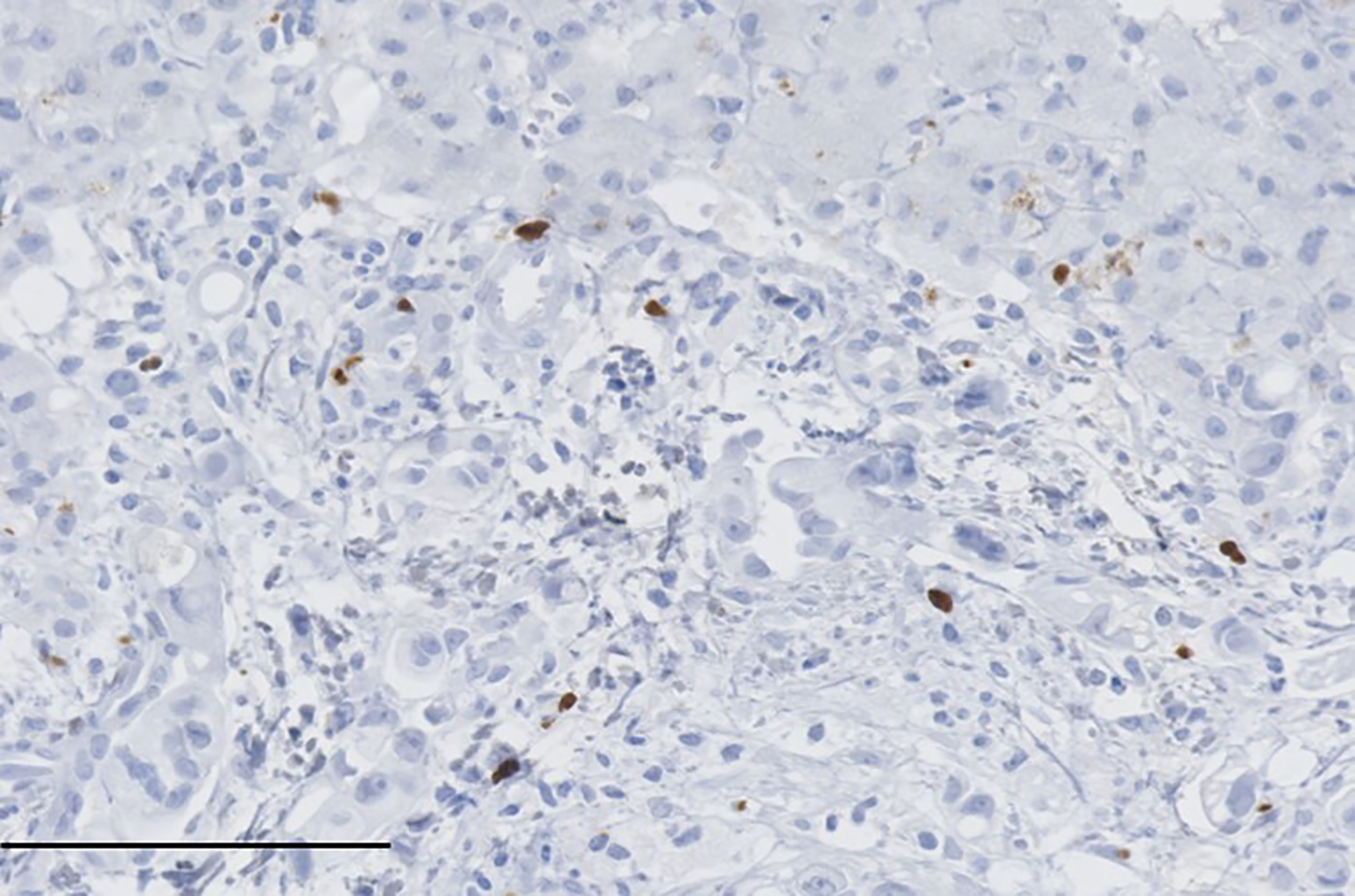

Supplement: Supplementary file 8 — FigS2D [file CAM4-9-3130-s008.tif]

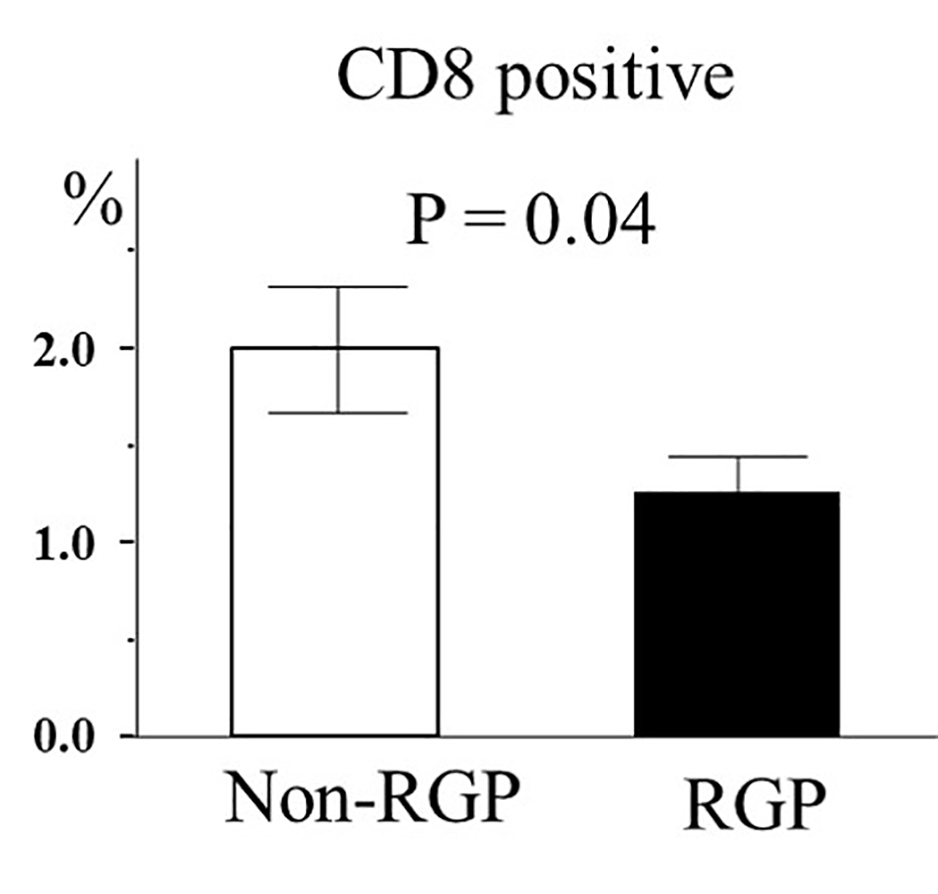

Supplement: Supplementary file 9 — FigS2E [file CAM4-9-3130-s009.tif]

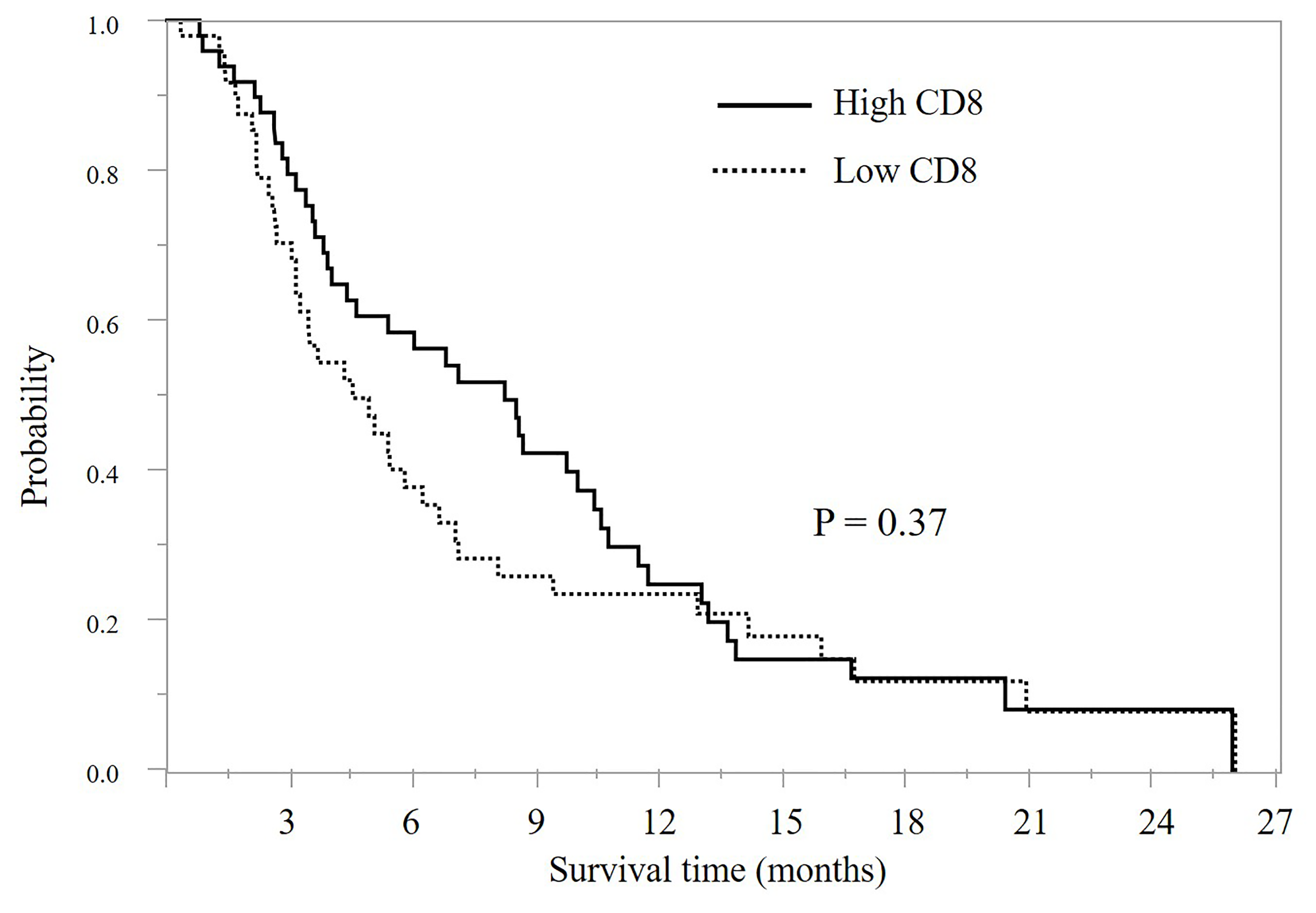

Supplement: Supplementary file 10 — FigS2F [file CAM4-9-3130-s010.tif]
