# Supplementary material for: The “histological replacement growth pattern” represents aggressive invasive behavior in liver metastasis from pancreatic cancer
Source: Cancer Med. 2020 Mar 5;9(9):3130–41. doi: 10.1002/cam4.2954 (PMC7196051; doi:10.1002/cam4.2954)
Supplement: Supplementary file 11 — TableS1‐S2 [file CAM4-9-3130-s011.docx]

| **Supplementary Table S1: Antibodies and immunohistochemical assays** | | | | | | |  |  |
| --- | --- | --- | --- | --- | --- | --- | --- | --- |
| Marker | Source | Type | Clone | Procedure | Dilution | Antigen retrieval | Visualization | Manufacturer |
| CD4 | Rabbit | Monoclonal | SP35 | Autostainer | Ready-to-use | Heat (95°C, 64 min.),CC1: EDTA buffer (pH 8.5) | iVIEW DAB Detection Kit | Ventana(Tucson, AZ, USA) |
| CD8 | Rabbit | Monoclonal | SP57 | Autostainer | Ready-to-use | Heat (95°C, 64 min.),CC1: EDTA buffer (pH 8.5) | ultra View Universal DAB Detection Kit | Ventana |
| FOXP3 | Mouse | Monoclonal | 236A/E7 | Manual | 1:500 | A/C (121°C, 10 min.),Citrate buffer (pH 9.0) | Standard DAB procedure | Abcam(Cambridge, UK) |
| Abbreviations: M/W, microwave; EDTA, ethylenediaminetetraacetic acid; DAB, diaminobenzidine. For FOXP3, slides were dewaxed and rehydrated in distilled water, and endogenous peroxidase activity was then blocked by immersion in 3% hydrogen peroxide in methanol for 10 minutes. After antigen retrieval, the slides were incubated overnight at 4°C with each primary antibody. The slides were then further incubated with anti-mouse secondary antibody (EnVision+ System-HRP Labelled Polymer Anti-mouse, Dako, Tokyo, Japan) for FOXP3, and staining was detected using a standard diaminobenzidine procedure. Finally, the sections were counterstained with hematoxylin. | | | | | | | | |

| **Supplementary Table S2** Suvival analysis of chemotherapy by rejimen | | | | | |
| --- | --- | --- | --- | --- | --- |
|  |  |  | Overall survival | | |
| Regimen | Growth pattern | n (%) | Median (range) | HR (95%CI) | P-value |
| GnP/FOLFIRINOX | Non replacement | 12 (11) | 13.0 (6.8-not reached) | 1.0 |  |
|  | Replacement | 14 (13) | 4.5 (2.8-8.2) | 3.6 (1.29-14.2) | 0.01 |
|  |  |  |  |  |  |
| Others | Non replacement | 41(38) | 8.6 (5.1-12.9) | 1.0 |  |
|  | Replacement | 40 (38) | 3.5 (2.7-5.4) | 2.5 (1.52-3.99) | ＜0.01 |
| HR;Hazard Ratio CI;Confidence interval | | | | | |
