# Supplementary material for: The “histological replacement growth pattern” represents aggressive invasive behavior in liver metastasis from pancreatic cancer
Source: Cancer Med. 2020 Mar 5;9(9):3130–41. doi: 10.1002/cam4.2954 (PMC7196051; doi:10.1002/cam4.2954)
Supplement: Supplementary file 12 — figure legends [file CAM4-9-3130-s012.docx]

**Supplementary figure S1. Photomicrographs of biopsied liver metastasis showing the Desmoplastic GP and Unclassified GP (hematoxylin and eosin staining)**

Desmoplastic GP is represented in (a,b) and unclassified GP is represented in (c,d) with hematoxylin and eosin staining. The region in the black box in (a,c) is shown in (b, d). In the desmoplastic GP, nests of tumor cells were separated from the liver parenchyma by a layer of desmoplastic stroma, with no direct contact between the tumor cells and liver parenchyma. Inflammatory cells infiltrate is present in the stroma near by the invasive front. In the unclassified GP, some area of tumor invasive front showed the replacement GP but inflammatory cells were infiltrated at the tumor-liver interface and separated tumor from liver parenchyma. The predominant GP in this section was classified into unclassified GP.

T, tumor; L, liver parenchyma; Ds, desmoplastic stroma; original magnification: (a, c) 10×, (b, d) 40×, Bar, 100 μm.

**Supplementary figure S2. Immunohistochemical features of infiltrating inflammatory cells stained by CD4, CD8 and FOXP3 and prognostic impacts.**

The representative photos are shown: (a) hematoxylin and eosin staining. The region in the black box in (a) is shown in (b,c,d). (b) CD4, (c) CD8, (d) FOXP3.

T, tumor; L, liver parenchyma; original magnification: (a) 10×, (b,c,d) 40×, Bar, 100 μm

A comparison of positive percentage of CD8 at the periphery of LM and tumor-liver interface is shown in (e). Kaplan–Meier survival curves showing comparison of overall survival between CD8 high and CD8 low groups is shown in (f).
